# Integrated Multi-Omics Analysis Reveals ACO2 as an Oxidative-Stress-Related Biomarker in Alzheimer’s Disease

**DOI:** 10.3390/biom16101435

**Published:** 2026-10-02

**Authors:** Wenbo Dou, Xue Chen, Hang Hong, Qiong Liu, Sa Xiao, Lian Li, Jiancheng Yu, Chunlan Tang

**Affiliations:** 1 School of Public Health, Health Science Center, Institute of One Health Science, Ningbo University, Ningbo 315211, China; 2411100005@nbu.edu.cn (W.D.); 2601140256@nbu.edu.cn (X.C.); honghang@nbu.edu.cn (H.H.); liuqiong@nbu.edu.cn (Q.L.); 2411140304@nbu.edu.cn (S.X.); ll19857840971@163.com (L.L.); 2 Faculty of Electrical Engineering and Computer Science, Ningbo University, Ningbo 315211, China; 3 Zhejiang Engineering Research Center of Advanced Mass Spectrometry and Clinical Application, Institute of Mass Spectrometry, Ningbo University, Ningbo 315211, China

**Keywords:** Alzheimer’s disease, oxidative stress, ACO2, proteomics, single-cell analysis, WGCNA

## Abstract

Alzheimer’s disease (AD) is a progressive neurodegenerative disorder characterized by cognitive decline and memory impairment. Oxidative stress plays an important role in disease progression in AD. However, the molecular mechanisms underlying oxidative stress in AD remain unclear and reliable biomarkers for its detection have not been identified. In this study, bioinformatics analyses integrating differential expression analysis and weighted gene co-expression network analysis were performed to identify oxidative-stress-related differentially expressed genes. Using machine learning algorithms, *ACO2* was identified as a significantly downregulated hub gene associated with AD. Single-cell transcriptomic analysis further showed markedly reduced *ACO2* expression levels in hippocampal neurons of patients with AD. To validate these findings, proteomic analysis, quantitative real-time polymerase chain reaction (qPCR), and Western blotting were performed in a mouse model of AD. These results confirmed decreased *ACO2* mRNA and protein expression levels in the hippocampus. Consistently, qPCR analysis of peripheral blood samples from patients with AD revealed significantly reduced *ACO2* expression levels compared to those in healthy controls. Collectively, these findings identified *ACO2* as an oxidative-stress-related biomarker associated with AD and suggested its potential value as an AD-related candidate biomarker.

## 1. Introduction

In Alzheimer’s disease (AD), the brain degenerates slowly over time. This mostly affects memory and cognitive abilities, which can significantly impact quality of life [1]. Currently, more than 50 million people globally have AD, and this number is expected to double to 152 million by 2050 [2]. The causes of AD are complicated and not yet fully understood, and they involve several risk factors, signaling pathways, and molecular mechanisms. The consensus is that AD is mostly caused by two factors: the build-up of a protein known as β-amyloid (Aβ) and the existence of neurofibrillary tangles, which arise from tau proteins undergoing abnormal phosphorylation [3,4,5]. Current treatments for AD mainly focus on easing the symptoms and slowing disease progression. However, because the fundamental biological processes driving this disorder are not fully understood, truly curative therapies have not been achieved [6]. Therefore, advancing our understanding of the pathogenesis of AD, improving its early diagnosis, and developing effective interventions have significant clinical and societal value.

Oxidative stress (OS) refers to the accumulation of reactive oxygen species (ROS) beyond what the antioxidant defense machinery can effectively manage, leading to biological stress [7]. Excessive ROS levels can damage DNA, RNA, and lipids. Damage to brain cells disrupts normal neuronal function and adversely affects memory, learning, and cognitive abilities [8,9]. Furthermore, OS induces molecular, endoplasmic reticulum, and mitochondrial damage, while promoting the accumulation of senescent cells. This triggers an inflammatory response, which leads to a cascade of metabolic, physiological, and pathological alterations. These physiological changes have a substantial effect on the initiation and development of various diseases that are often linked to the aging process, including neurodegenerative disorders, cardiovascular diseases, sarcopenia, osteoarthritis, and different forms of cancer [10,11,12,13]. Although oxidative stress is closely linked to metabolism, immunity, neural signaling, and aging processes, previous studies have predominantly focused on individual aspects and have lacked systematic integration and multi-omics analysis (e.g., transcriptomics and proteomics). This limitation has hindered a comprehensive understanding of the global mechanisms involved in physiological and pathological processes.

The primary hypotheses for the pathogenesis of AD include amyloid-beta, tau protein, inflammation, and metal ions. The literature indicates that while these mechanisms operate independently, they are all associated with OS, which may serve as a pivotal link connecting these pathways [14]. OS plays a major role in the development of the symptoms of AD. Substantial evidence indicates that extensive oxidative damage occurs in the brain of patients with AD. It has been shown that the build-up of Aβ can increase the production of ROS, which in turn, triggers OS. This can lead to neuronal apoptosis and disruption of both the structural integrity and functional activity of brain tissue. In turn, OS further promotes Aβ production, creating a positive feedback loop that exacerbates disease progression. Accumulating evidence has revealed a close association between OS and abnormal tau aggregation. OS can accelerate tau aggregation by promoting oxidation of essential fatty acids, thereby facilitating the formation of neurofibrillary tangles (NFTs) [15,16,17]. Furthermore, mitogen-activated protein kinase (MAPK), a critical kinase involved in tau phosphorylation, is activated under oxidative stress conditions [18]. Activation of this signaling pathway leads to tau hyperphosphorylation, increasing its propensity to assemble into PHFs and, eventually, NFTs. Recent advances have highlighted the role of OSRGs in AD pathology. The aberrant expression of these genes contributes to disease progression by modulating cellular antioxidant defenses, energy metabolism, and mitochondrial function, thereby aggravating neurodegenerative changes. For instance, mice deficient in *SOD2* (mitochondrial manganese superoxide dismutase) exhibit increased mitochondrial OS and increased tau phosphorylation, suggesting a common regulatory role for SOD family members in tau modification [19]. Likewise, the loss of the mitochondrial deacetylase sirtuin-3 (*SIRT3*) results in neuronal hyperexcitability, decreased viability, and increased OS [20]. Our findings thus far show that OS is a key factor in initiating the development and promoting the progression of AD. Nevertheless, the ways in which OSRGs may be leveraged for the diagnosis of AD is not well understood and requires further investigation.

In recent years, the use of bioinformatics has become increasingly important for identifying disease-related biomarkers and exploring the underlying mechanisms of diseases. Publicly accessible transcriptomic repositories, such as the Gene Expression Omnibus (GEO), contain a large number of disease-associated gene expression profiles and provide valuable data resources for screening differentially expressed genes (DEGs) and analyzing molecular alterations [21]. Based on these datasets, weighted gene co-expression network analysis (WGCNA) can be used to characterize global gene expression patterns, identify co-expression modules closely related to disease phenotypes, and screen for potential core regulatory genes. Machine learning methods have been increasingly applied to optimize and refine candidate feature genes, whereas receiver operating characteristic (ROC) curve analysis is commonly used to preliminarily evaluate diagnostic performance [22]. In addition, single-cell transcriptomic analysis enables the investigation of gene expression differences at the cell-type level, offering new insights into the cellular origin and potential biological roles of core genes [23].

Given the lack of systematic analyses targeting OS in AD, the purpose of this study was to screen for OS-related DEGs (OSRDEGs) in AD using multi-omics analysis, WGCNA methods, and machine learning algorithms. Additionally, the functional enrichment pathways of the core genes, their roles in immune cell infiltration, and their expression in single cells were investigated. Furthermore, an APP/PS1 transgenic mouse model was used to validate the expression levels of core genes using proteomics, quantitative polymerase chain reaction (qPCR), and Western blotting. Finally, the core genes identified in the above screening and validation procedures were measured in the peripheral blood samples of patients with AD to determine their clinical diagnostic value. This study aimed to identify potential OS biomarkers of AD and comprehensively elucidate the molecular pathological mechanisms associated with OS in AD. This will lay the groundwork for the early diagnosis of AD.

## 2. Materials and Methods

### 2.1. Materials

The RNAprep Pure Tissue Kit (DP431) was purchased from TianGen Biotech (Beijing, China). The TransZol Up Simple RNA Kit was purchased from TransGen Biotech (Beijing, China). TransScript All-in-One First-Strand cDNA Synthesis SuperMix (with gDNA removal agent), PerfectStart^®^ Green qPCR SuperMix, and Easy II Protein Quantification Kit were purchased from Beijing Quanshijin Biotechnology (Beijing, China). The anti-ACO2 (1:6000) and GAPDH (1:100,000) antibodies were sourced from Wuhan Sanying Biotechnology (Wuhan, China). Horseradish-peroxidase-labeled goat anti-rabbit IgG and goat anti-mouse IgG (1:10,000) were provided by Shanghai Ober Bio-Technology (Shanghai, China), and the bicinchoninic acid assay kit was purchased from Biyun Tian Biotechnology (Shanghai, China). The proteomics-related reagents and instruments are listed in Supplementary File S2 (Tables S13 and S14).

### 2.2. Animals

All methodologies involving animals in this study received the requisite approval from the Animal Ethics and Welfare Committee of Ningbo University. The mice were sourced from Hangzhou Ziyuan Laboratory Animal Co., Ltd. (Hangzhou, China). Twelve-month-old male wild-type (WT) and APP/PS1 mice (*n* = 10 per group) were maintained in a standardized, specific-pathogen-free environment at the Ningbo University Laboratory Animal Center under controlled environmental conditions. The animals were provided with unrestricted access to food and water throughout the 7-day adaptation phase. After acclimatization, the mice were anesthetized with isoflurane and euthanized using cervical dislocation. The brain was rapidly removed and the hippocampal tissue was carefully dissected on ice. The collected hippocampal samples were immediately stored at −80 °C until further analysis.

### 2.3. Human Populations

Twenty-four patients with AD were recruited from the Affiliated Kangning Hospital of Ningbo University and 24 healthy control (CTL) participants were recruited from the Ningbo community. Peripheral blood samples were collected. Patients in the AD group ranged in age from 55 to 87 years. The CTL participants were matched to the patients in terms of age and sex, and had no history of AD.

### 2.4. RNA Extraction and qPCR Analysis

Total RNA was extracted from mouse hippocampal tissues and human peripheral blood samples using commercial RNA extraction kits according to the manufacturer’s instructions. The extracted RNA was reverse-transcribed into cDNA. Detection was performed using the LightCycler^®^ 480 II real-time PCR system (Roche, Basel, Switzerland). GAPDH was utilized as the standard against which relative quantities were calculated using the 2^−ΔΔCt^ method. Each sample was analyzed in triplicate. The primer details are provided in Supplementary File S2 (Table S12).

### 2.5. Western Blotting Analysis

Protein samples were adjusted to 2.5 µg/µL with 5× sodium dodecyl sulfate (SDS) loading buffer and heated at 95 °C for 5 min. Ten microliters of each sample were loaded onto a 10% SDS-polyacrylamide gel to separate the proteins, which were then transferred to polyvinylidene fluoride membranes. The membranes were blocked using 5% skim milk at room temperature for 1 h and then incubated with primary antibody at 4 °C overnight. After washing with TBST, the membranes were incubated with secondary antibodies diluted 1:10,000 for 1.5 h at room temperature. A Tanon 5200 chemiluminescence imaging system was used to visualize the protein signals and ImageJ (version 1.54p) was used to measure band intensities. The target protein expression levels were normalized to GAPDH levels. The ACO2/GAPDH band intensity ratio was used for statistical analysis. Quantification was based on independent biological replicates, with each biological replicate representing an individual animal (*n* = 3 per group). Densitometric analysis was performed by an investigator blinded to the group allocation.

### 2.6. Data Source of Bioinformatics Analysis

Two microarray datasets related to AD, GSE5281 [24] and GSE1297 (training set) [25], were obtained from the GEO repository. The GSE5281 dataset was sourced from the GPL570 array platform and comprises 10 AD and 13 CTL samples, whereas the GSE1297 dataset was sourced from the GPL96 array platform and comprises 22 AD and 9 CTL samples. An AD-related high-throughput dataset, GSE173955 (validation set) [26], based on the GPL18460 platform and comprising 8 AD and 10 CTL samples, and the GSE175814 dataset [27], which provides information on single-cell sequences from two patients with AD and two CTL individuals, were also used. All data were derived from hippocampal tissue. We also identified OS-related genes (OSRGs) from GeneCards, selecting 2179 genes with correlation scores ≥ 7 for subsequent analysis (Supplementary File S1 (Table S3)) (Supplementary File S2 (Figure S4)) [28,29,30].

### 2.7. Identification Differentially Expressed Genes of AD and Functional Enrichment Analysis

The two datasets were independently pre-processed prior to integration. For GSE5281, the probe IDs were mapped to gene symbols using the hgu133plus2.db annotation package, whereas for GSE1297, the hgu133a.db annotation package was used. When multiple probes were mapped to the same gene, the probe with the highest mean expression level within each dataset was retained as the representative probe for that gene. Subsequently, log2 transformation was performed when necessary, according to the distribution of expression values, and each dataset was independently normalized using the normalizeBetweenArrays function in the limma package. After probe annotation, only the genes shared by both platforms were retained for subsequent integration analysis. The GSE1297 and GSE5281 datasets were then integrated using the R package “limma” (version 3.62.2) [31], and batch effects between the two studies were removed using the removeBatchEffect function. DEGs were identified using the thresholds of |log_2_ fold change (FC)| > 0.5 and an adjusted *p*-value < 0.05. Functional enrichment analyses of the DEGs were performed using the R package “clusterProfiler” (version 4.14.4) [32]. The Gene Ontology (GO) enrichment analysis included three major categories: biological processes (BP), cellular components (CC), and molecular functions (MF). Kyoto Encyclopedia of Genes and Genomes (KEGG) pathway enrichment analysis was performed to identify significantly enriched pathways. Differences were considered statistically significant at an adjusted *p*-value < 0.05.

### 2.8. Analysis of Gene Expression Patterns and Co-Expression Modules

WGCNA was carried out using the R package “WGCNA” (version 1.73) [33]. All genes in the merged dataset were used to construct the co-expression networks. A cluster tree diagram was constructed to detect outliers and remove abnormal samples. The optimal soft threshold power, β, was chosen based on the rules for a roughly scale-free structure to ensure the network’s scale-free characteristics. The smallest module that can be used is one containing at least 30 genes. We found gene modules from the topological overlap matrix (TOM) by using “average linkage hierarchical clustering” and defining module boundaries using “dynamic tree cutting”. Similar modules were joined using a threshold of 0.25. Subsequently, we derived the MEs by extracting the first principal component of each module and assessed their relationships with clinical features using Pearson’s correlation, allowing us to identify the modules most associated with AD. After obtaining module information from the dataset, it was split into groups. One group was designated the CTL, and the WGCNA package’s built-in function “modulePreservation” [34] was used to perform WGCNA module conservation analysis.

### 2.9. Identification of OSRDEGs in AD

DEGs were cross-analyzed with genes from WGCNA and OSRGs via Venn diagrams to identify OSRDEGs. In addition, enrichment analyses were carried out to ascertain the biological functions and signaling pathways correlated with the OSRDEGs. To identify candidate hub genes, three complementary machine learning algorithms, including RF, LASSO regression, and SVM-RFE, were applied to the OSRDEGs. RF analysis was performed using the R package “randomForest” (version 4.7), and gene importance was evaluated according to the MeanDecreaseGini metric. LASSO regression was conducted using the R package “glmnet” (version 4.1) [35], with five-fold cross-validation applied to determine the optimal regularization parameter (λ); genes with non-zero regression coefficients were retained as candidate features. SVM-RFE was performed using the R package “e1071” (version 1.7) [36], with five-fold cross-validation combined with recursive feature elimination to identify the optimal feature subset. The results obtained from RF, LASSO, SVM-RFE, and MCODE analyses were integrated to identify the core candidate gene, *ACO2*. The discriminatory performance of *ACO2* for distinguishing AD from control samples was evaluated using ROC analysis implemented with the R package “pROC” (version 1.18.5). *ACO2* expression was used as the predictor and sample group as the outcome variable. The area under the ROC was calculated to quantify discriminatory performance, and the corresponding 95% CI was estimated using the DeLong method.

### 2.10. Gene-Specific Enrichment Analysis

GSEA [37] of hub genes was carried out using the gseKEGG function in the R package “clusterProfiler”. Peaks on the upper-left curve represent active functions or pathways in the higher-expression group of hub genes, whereas peaks on the lower-right curve indicate active functions or pathways in the lower-expression group. The top five and bottom five pathways ranked by enrichment scores were selected.

### 2.11. Analyzing Immune Infiltration

We used the CIBERSORT (version 0.1.0) [38] method to analyze the gene expression matrix of our samples, estimating the proportion of 22 different immune cell subtypes. The association between hub genes and immune cell infiltration was investigated using Spearman’s correlation analysis.

### 2.12. Single-Cell RNA Sequencing (ScRNA-seq) Analysis

The 10× datasets were switched to Seurat format and analyzed using the R package Seurat (version 5.2.1) [39]. Cells were removed when mitochondrial gene expression surpassed 5%, nCount_RNA fell outside the 1000–20,000 range, and nFeature_RNA was outside 200–5000. Normalization and scaling were performed using the SCTransform function. Cell clustering was manually annotated based on the published literature.

## 3. Results

### 3.1. The Screening of DEGs in AD and Functional Enrichment Analysis

**Figure 1:**
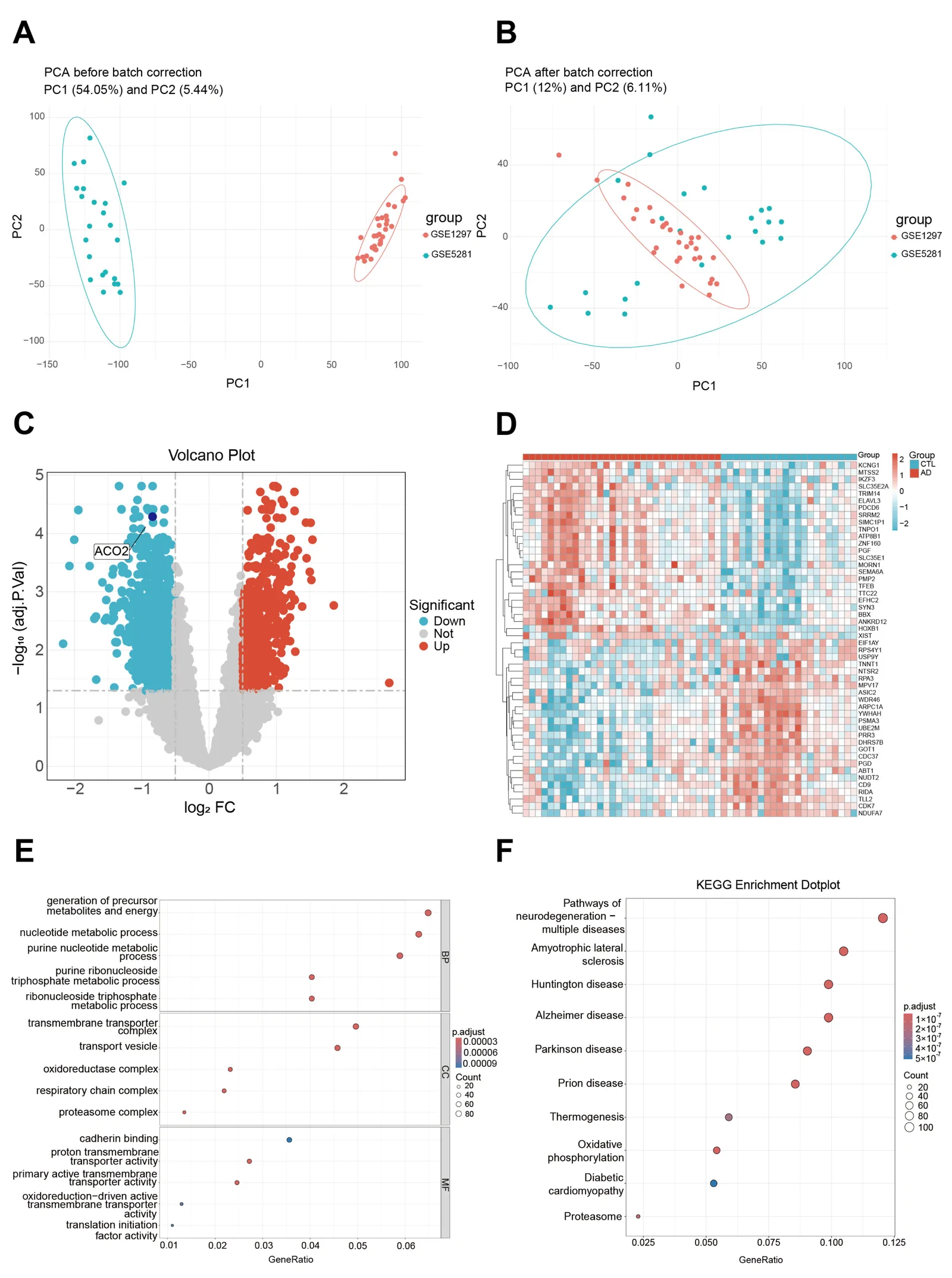
Identification of AD DEGs. (**A**) PCA plot before batch-effect correction. (**B**) PCA plot after batch effect rectification. (**C**) Volcano plot of DEGs. (**D**) Heatmap of DEGs, showing the top 50 genes. (**E**) GO enrichment analysis of DEGs. (**F**) KEGG enrichment analysis of DEGs.

PCA revealed that the GSE1297 and GSE5281 datasets were clearly divided in the principal component space because of batch effects, indicating that batch variation influenced the data analysis. Therefore, batch correction was performed. The distributions of the two datasets after correction became more closely aligned with increased overlap, demonstrating that the batch effects were effectively removed, and data comparability was significantly improved (Figure 1A,B). In total, 1611 DEGs were detected, comprising 575 genes with increased expression levels and 1036 with decreased expression levels (Figure 1C) (Supplementary File S1 (Table S1)). To further evaluate the robustness of the results with respect to the data integration strategy, we performed a sensitivity analysis (Supplementary File S2 (Figure S3)). The heatmap shows 50 genes with the most pronounced differences in expression level (Figure 1D). Functional enrichment analysis showed that BP category mainly comprised precursor metabolism, energy metabolism, nucleotide metabolism, and purine nucleotide metabolism; CC included transmembrane transporters, transport vesicles, and oxidoreductase complexes; and MF included cadherin binding and major active transmembrane transporter activity (Figure 1E). KEGG analysis identified major pathways, including AD, oxidative phosphorylation, and the neurodegenerative disease pathway (Figure 1F).

### 3.2. WGCNA Identified Key Gene Modules Associated with AD

**Figure 2:**
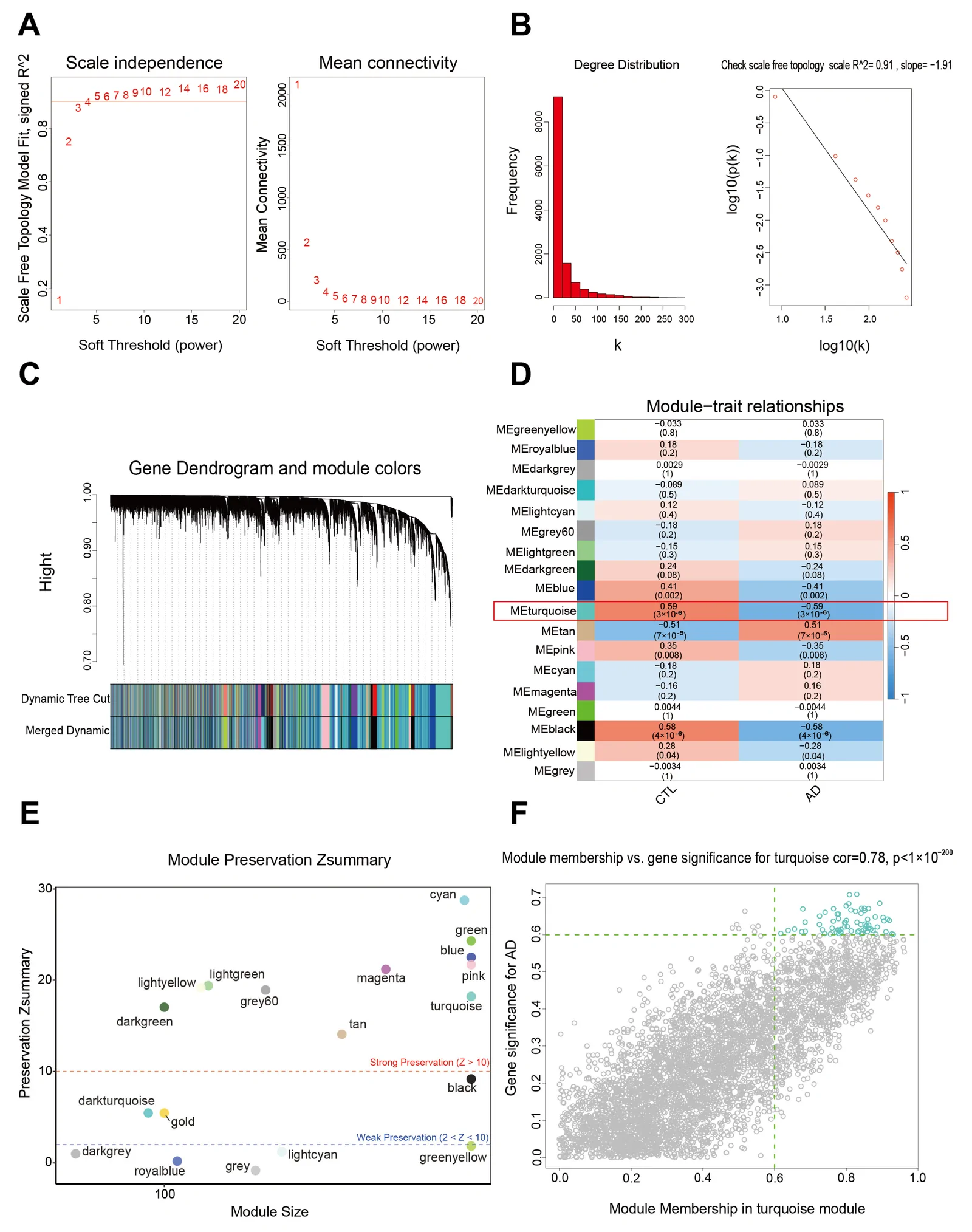
Finding the key genes linked to AD. (**A**) Choosing the best power for thresholding. (**B**) Connectivity distribution histogram along with the scale-free topology fit when β = 6 (R^2^ = 0.91). (**C**) Hierarchical dendrogram of intergenic clustering relationships. (**D**) Heatmap of correlations between eigengenes and clinical characteristics. (The red box indicates the module showing the strongest correlation with the clinical characteristics. Red and blue indicate positive and negative correlations, respectively, with color intensity reflecting the magnitude of the correlation coefficient.) (**E**) WGCNA module retention analysis. (**F**) Relationship between gene significance and module membership in the turquoise module.

WGCNA was performed on CTL and AD samples to identify phenotype-related genes in AD. A power of 6 was used to create a topology similar to a scale-free topology, which was validated using the topology fit index and connectivity results (Figure 2A). An R^2^ value of 0.91 indicated an excellent fit, and the degree distribution of the network largely followed a power-law relationship, further supporting its scale-free topology (Figure 2B). Eighteen distinct gene modules were identified by constructing a hierarchical clustering dendrogram using a gene correlation matrix (Figure 2C). The “turquoise” module demonstrated the most meaningful link with the AD phenotype (Cor = −0.59, *p* = 3 × 10^−6^), making it the clinically most relevant module (Figure 2D). Conservation analysis revealed that the turquoise module resides in a highly conserved region (Z > 10), featuring a moderate module size and strong biological stability (Figure 2E). The scatter plots showed a strong positive association between GS and MM in the turquoise module (Cor = 0.78, *p* < 1 × 10^−200^), indicating high consistency between module genes and AD-relatedness. Accordingly, 71 genes that satisfied the requirements of GS > 0.6 and MM > 0.6 were chosen for analysis (Figure 2F) (Supplementary File S1 (Table S2)).

### 3.3. Screening of OSRDEGs and Functional Enrichment Analysis

**Figure 3:**
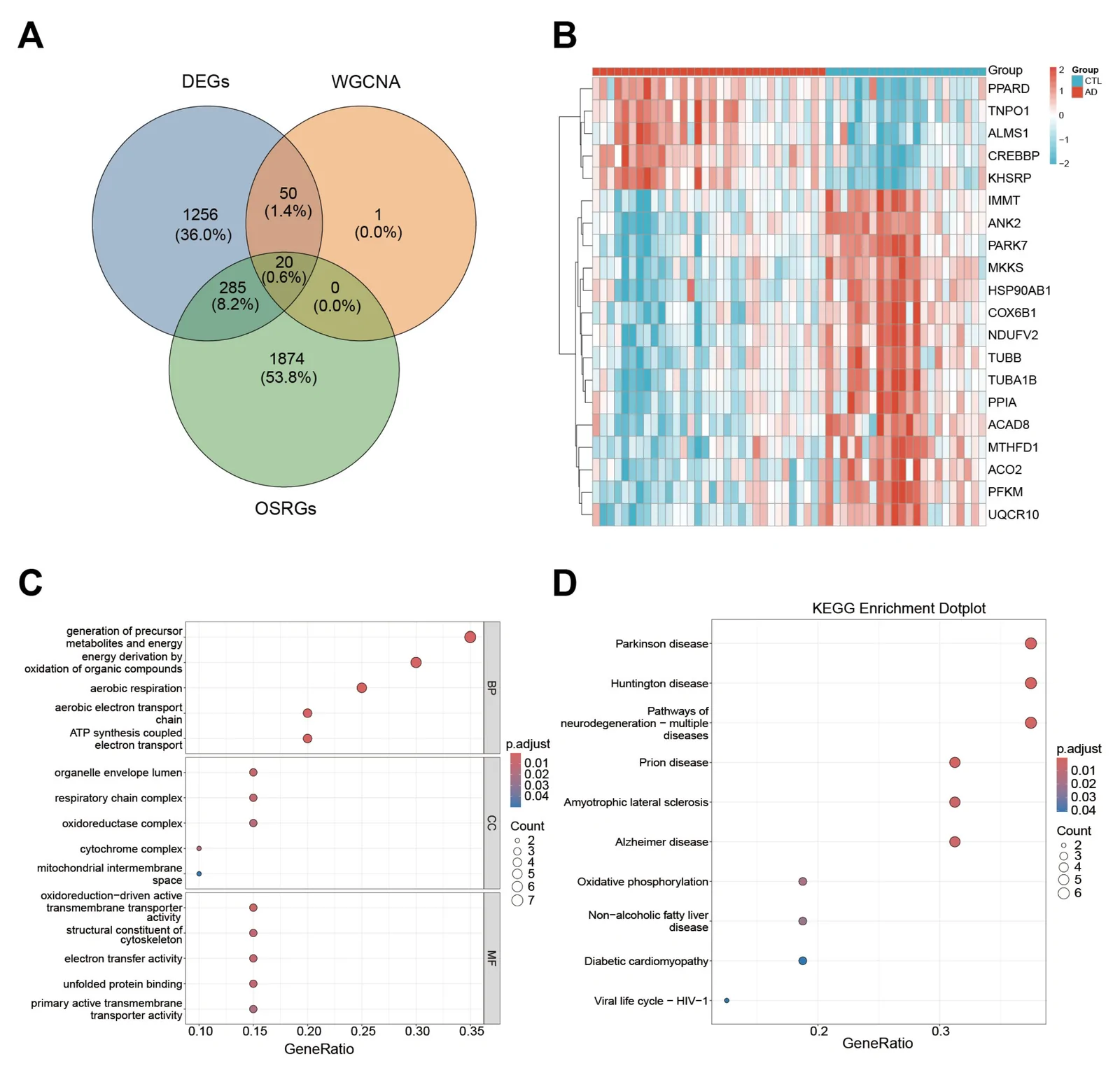
Screening OSRDEGs and functional screening analysis. (**A**) Venn Diagram of DEGs, genes from WGCNA, and OSRGs. (**B**) Heatmap of OSRDEGs expression in AD and CTL samples. (**C**) Functional categories enriched by the OSRDEGs based on GO analysis. (**D**) OSRDEG-associated pathways identified through KEGG analysis.

The intersection of 1611 DEGs, 71 genes from the turquoise module in the WGCNA, and 2179 OSRGs was performed to obtain 20 OSRDEGs (Figure 3A) (Supplementary File S1 (Table S7)). Subsequently, a heat map was used to visualize the differences in the expression levels of these genes (Figure 3B). Analysis of these 20 genes showed that they were primarily enriched in precursor metabolism, energy production, oxidation of organic compounds for energy in the BP category; organelle membrane compartments and redox enzyme complexes in the CC category; and redox-driven transmembrane transport activity in the MF category (Figure 3C). KEGG analysis indicated that the significant pathways were multiple neurodegenerative-disease-related pathways and oxidative phosphorylation (Figure 3D).

### 3.4. Identification of Hub Genes Through Machine Learning and MCODE Analysis

**Figure 4:**
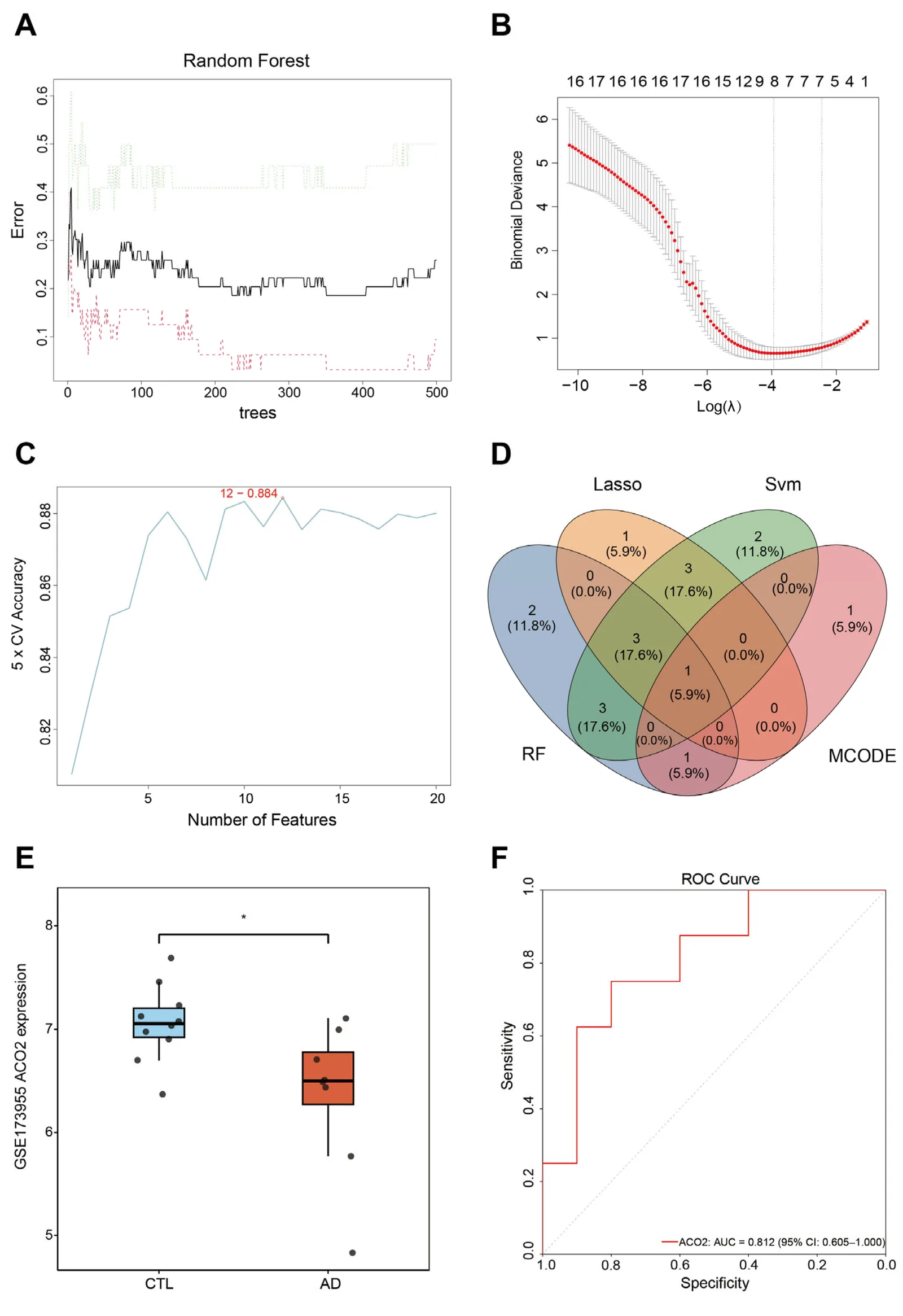
Identification of hub OSRDEGs using machine learning and MCODE analysis. (**A**) The RF algorithm error varies depending on the number of trees. (**B**) LASSO regression cross-validation results curve. (**C**) Cross-validation accuracy curve of the SVM algorithm with different numbers of features. (**D**) The genes shared by the three machine learning tools and the MCODE analysis. (**E**) Expression of *ACO2* in the GSE173955 validation set. (**F**) ROC of *ACO2* in the GSE173955 validation dataset. * *p* < 0.05.

Hub genes among the 20 OSRDEGs were further screened by integrating machine learning tools with the MCODE plugin in Cytoscape (version 3.9.1). The RF algorithm was employed to identify the 10 most influential genes (Figure 4A). Subsequently, feature selection based on the LASSO regression algorithm identified eight candidate genes (Figure 4B). Concurrently, the SVM-RFE algorithm identified 12 optimal feature genes and achieved the highest model accuracy of 0.884 (Figure 4C) (Supplementary File S1 (Tables S4–S6)). MCODE module analysis further filtered out three core genes. Gene sets from the above four methods revealed *ACO2* as the hub OSRDEG (Figure 4D). Subsequently, *ACO2* was further validated using the independent validation dataset GSE173955. Consistent with previous findings, *ACO2* expression levels were significantly lower in the AD group than in the CTL group (Figure 4E). Further, ROC analysis showed that *ACO2* achieved an AUC of 0.812 in the validation dataset (Figure 4F), suggesting its potential ability to discriminate between AD and CTL groups.

### 3.5. Single-Gene GSEA of ACO2-Related Pathways

**Figure 5:**
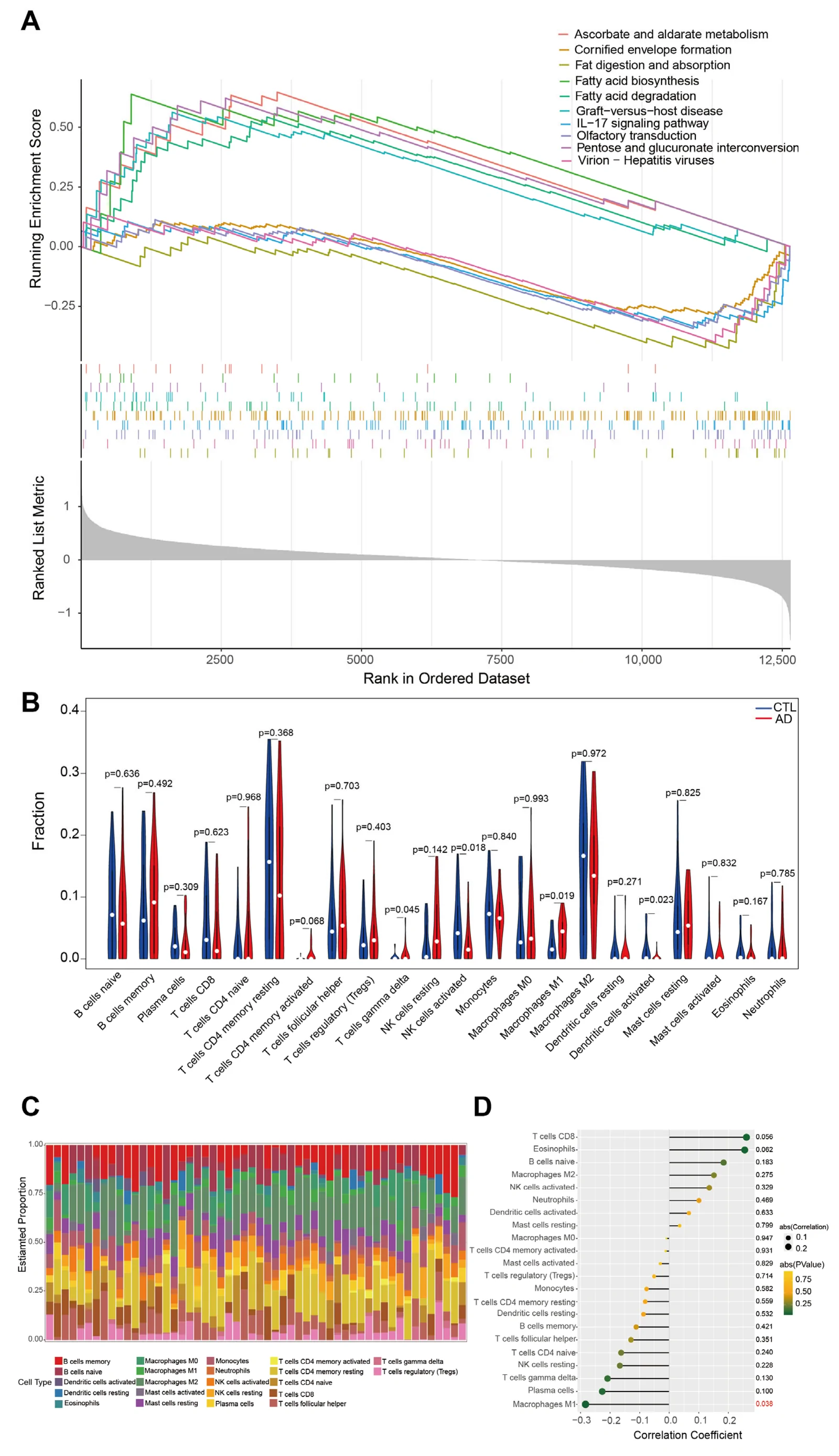
Single-gene GSEA and immune infiltration analysis results. (**A**) Single-gene GSEA reveals significantly enriched signaling pathways and biological processes. (**B**) Distribution patterns of 22 immune cell types across the AD and CTL. (**C**) Immune cell composition of each sample. (**D**) Lollipop plot illustrating the link between OSRDEGs and 22 immune cell types in AD and CTL.

Single-gene GSEA was performed to determine the link between *ACO2* levels and important biological pathways. A comparison of how gene sets are enriched between groups of high and low *ACO2* expression levels revealed that the group with higher *ACO2* expression levels showed significant enrichment in pathways including fatty acid biosynthesis, ascorbate and aldarate metabolism, and the IL-17 signaling pathway, suggesting that *ACO2* plays a crucial role in lipid metabolism, antioxidant metabolism, and immune response regulation (Figure 5A).

### 3.6. Analysis of Immune Cell Infiltration Revealed a Link Between ACO2 and Immune Cells

“CIBERSORT” was applied to estimate the levels of 22 immune cell populations. Individual differences in immune cell composition were observed, with T cells representing the largest proportion of infiltrating immune cells in the disease. Compared to the CTL group, the AD group showed significant upregulation of T-cell gamma delta and M1 macrophages, while activated NK cells and activated dendritic cells were significantly downregulated (Figure 5B,C). Moreover, the level of *ACO2* expression showed a clear link with M1 macrophages (Figure 5D).

### 3.7. Expression of ACO2 in Single Cells

**Figure 6:**
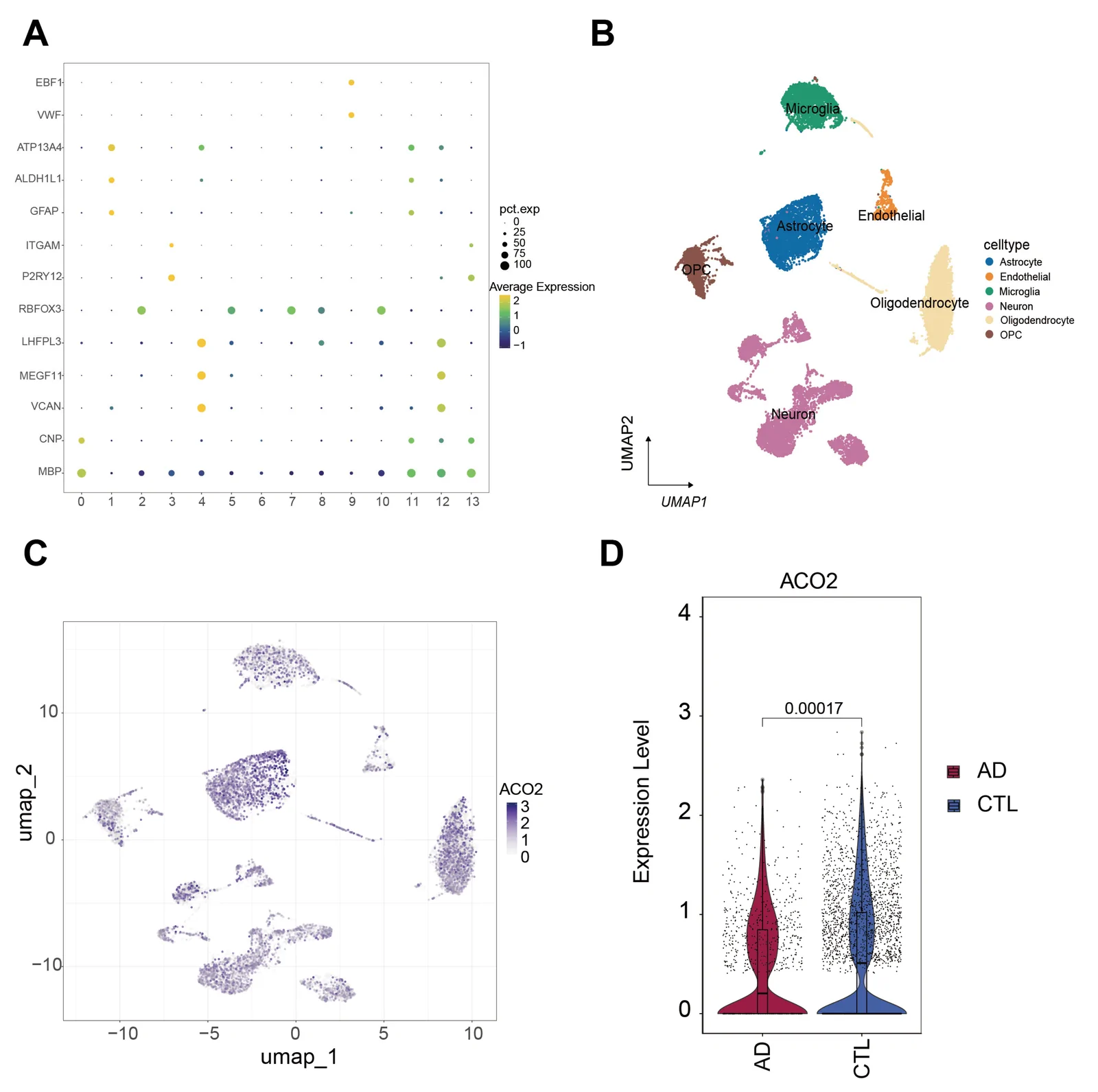
Results of Single-cell transcriptome analysis of AD and CTL. (**A**) Marked gene bubble diagram. (**B**) Single-cell transcriptome UMAP clustering plot. (**C**) Expression analysis of *ACO2* in the single-cell level. (**D**) The way *ACO2* is expressed in neurons.

ScRNA-seq datasets from the GEO, consisting of two CTL and two AD samples, were analyzed to explore *ACO2* expression across cell types. Cells were retained only when mitochondrial gene expression was below 5%, and filtering thresholds of 200–5000 for nFeature_RNA and 1000–20,000 for nCount_RNA were applied. This dataset contained 2000 genes. All highly variable genes are shown in red (Supplementary File S2 (Figure S1A–E)). Cells were clustered using the “FindCluster” function, yielding 13 clusters. The cells were labeled using marker genes for microglia (*P2RY12* and *ITGAM*), astrocytes (*GFAP*, *ALDH1L1*, and *ATP13A4*), endothelial cells (*VWF* and *EBF1*), neuronal cells (*RBFOX3* and *TUBB*), oligodendrocytes (*MBP* and *CNP*), and oligodendrocyte precursor cells (*VCAN*, *MEGF11*, and *LHFPL3*) (Figure 6A,B). Differential expression analysis demonstrated a considerably higher level of *ACO2* in cells from the CTL group than in those from the AD group (Supplementary File S1 (Table S9)). Further analysis at the cell-type level showed that *ACO2* exhibited higher expression levels in neuronal cells within the CTL group, demonstrating significant upregulation compared to the AD group (Figure 6C,D). In addition, to reduce the risk of pseudoreplication arising from the treatment of individual cells as independent biological replicates, we performed donor-level pseudobulk analysis. The results showed that *ACO2* remained downregulated in AD donors, consistent with the cell-level FindMarker analysis. However, because the single-cell dataset included only two AD donors and two CTL donors, the statistical power at the donor level was limited and the between-group difference did not reach statistical significance. The corresponding pseudobulk analysis results are presented in Supplementary File S1 (Table S10).

### 3.8. Single-Cell Analysis of Neuronal Cells

**Figure 7:**
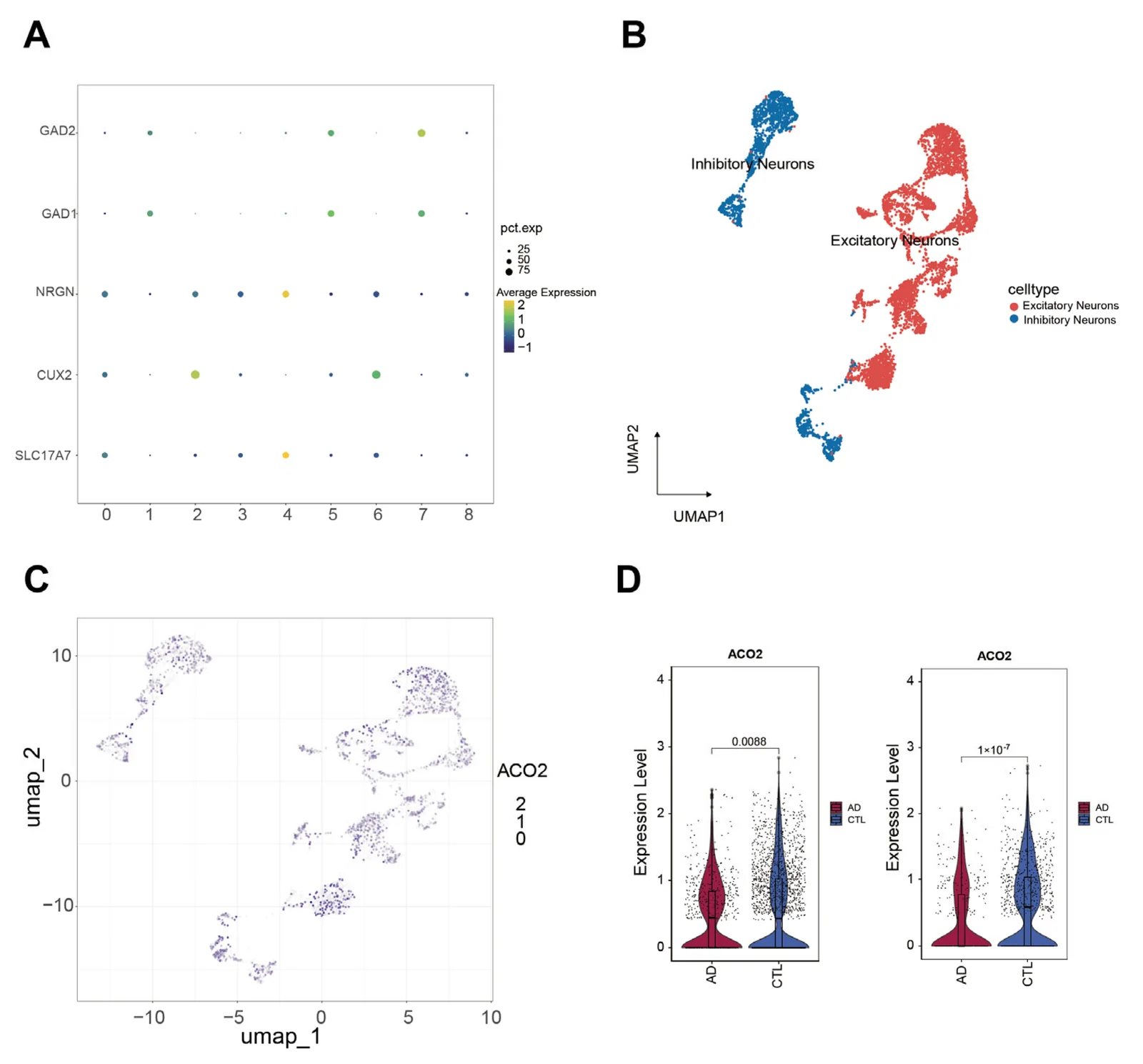
Subcluster identification and pseudo-time analysis. (**A**) Bubble plot of marker genes. (**B**) UMAP clustering diagram of neuronal subpopulations. (**C**) Expression Distribution of *ACO2* in Neuronal Subpopulations. (**D**) *ACO2* expression across different neuronal subtypes.

The neuronal cells were segmented and labeled using specific cell type marker genes for excitatory neurons (*SLC17A7*, *CUX2*, and *NRGN*) and inhibitory neurons (*GAD1* and *GAD2*) (Figure 7A–C), to gain deeper insights into *ACO2* expression within neuronal cells. Our findings indicated that the expression levels of *ACO2* in the two neuronal subtypes of AD were significantly lower than those in the CTL group (Figure 7D).

### 3.9. Experimental Validation of Key OSRDEGs in APP/PS1 Mouse Models

**Figure 8:**
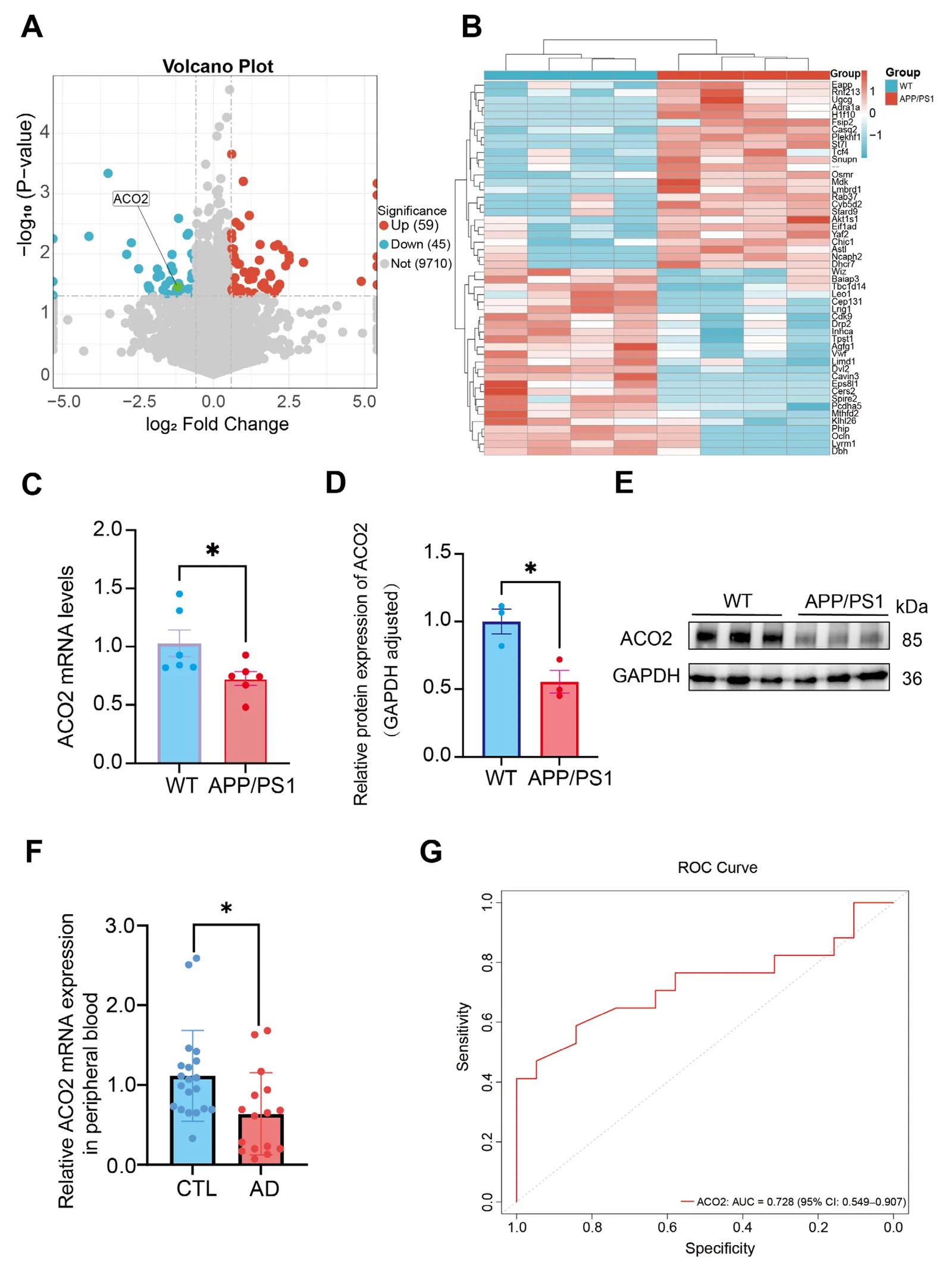
Experimental Validation Results of *ACO2*. (**A**) Volcano plot illustrating protein expression differences in the hippocampus between different mice. (**B**) Heatmap displaying hippocampal protein expression differences between different mice. (**C**) Comparison of *ACO2* mRNA expression levels between different mice, *n* = 6 per group. (**D**,**E**) Protein expression levels of ACO2 between different mice, *n* = 3 per group. (**F**) Comparison of peripheral blood *ACO2* mRNA expression between AD and CTL, *n* = 24 per group. (**G**) ROC of peripheral blood *ACO2* mRNA expression. * *p* < 0.05.

Proteomic analysis was performed to compare the differentially expressed proteins (DEPs) in hippocampal samples from WT and APP/PS1 mice (Supplementary File S2). A total of 104 DEPs were identified (screening criteria: FC ≥ 1.5 or ≤0.6667 and nominal *p* < 0.05; Benjamini–Hochberg correction was additionally applied for multiple testing, but no proteins remained significant at adjusted *p* < 0.05), including 59 upregulated proteins and 45 downregulated proteins, including ACO2 (Figure 8A) (Supplementary File S1 (Table S11)). A hierarchical clustering heatmap of DEPs in hippocampal tissues is shown in Figure 8B. GO analysis revealed that DEPs were predominantly enriched in processes associated with synaptic plasticity, learning and memory, transcriptional regulation, immune and inflammatory responses, and p53-mediated DNA-damage–induced intrinsic apoptotic signaling, along with abnormalities in receptor binding, endosomal recycling, and transition metal ion homeostasis (Supplementary File S2 (Figure S2A)). KEGG pathway analysis further demonstrated enrichment in key signaling pathways, including Notch, TGF-β, JAK-STAT, and Hippo pathways, as well as pathways related to synaptic vesicle cycling, lipid metabolism, and one-carbon metabolism (Supplementary File S2 (Figure S2B)). Collectively, these results indicate that DEPs in the hippocampus of APP/PS1 mice are involved in the widespread dysregulation of synaptic function, inflammatory responses, metabolic processes, and cellular signaling networks, which is consistent with typical AD pathology. Subsequently, qPCR and Western blotting analyses were performed to validate the proteomic findings. Data normality was first assessed using the Shapiro–Wilk test. As the qPCR data (Supplementary File S1 (Table S8)) showed a departure from normality, the differences between the WT and APP/PS1 groups were analyzed using a two-tailed Mann–Whitney U test. The results showed that *ACO2* mRNA expression levels were significantly reduced in the hippocampus of APP/PS1 mice compared to those in WT mice (Figure 8C). The Shapiro–Wilk test detected no significant deviation from normality in the Western blotting data; therefore, the ACO2/GAPDH densitometric ratios were compared using a two-tailed unpaired Welch’s t-test. The results showed that ACO2 protein expression levels were significantly lower in the hippocampus of APP/PS1 mice than in that of WT mice (Figure 8D,E).

### 3.10. Validation of Key OSRDEGs in Human Peripheral Blood Samples by qPCR

qPCR analysis was performed using peripheral blood samples from patients with AD and CTL subjects to further validate the changes in *ACO2* expression. The normality of the data was first assessed using the Shapiro–Wilk test. As the data were not normally distributed, differences between the AD and CTL groups were compared using a two-tailed Mann–Whitney U test. The results indicated that *ACO2* mRNA levels were significantly lower in subjects with AD than in healthy CTL subjects, which was consistent with the downregulation trend observed in the GEO dataset (Figure 8F). ROC analysis further showed that peripheral blood *ACO2* mRNA expression levels achieved an AUC of 0.728 for distinguishing AD from CTL groups (Figure 8G). These findings indicated that *ACO2* exhibits a consistently low expression level pattern in both public transcriptomic data and clinical peripheral blood samples, further supporting the potential value of *ACO2* as a candidate AD-related biomarker.

## 4. Discussion

AD is a degenerative condition that affects the brain, causing memory loss and problems with thinking and concentration. This places significant strain on public health and social care services [40]. OS is considered a key mechanism underlying the onset and progression of AD. It plays a role in the development of AD by inducing neuronal damage, disrupting synaptic structures, and exacerbating cellular dysfunction [41]. Although research in this area has been extensive, studies that systematically analyze OS-related molecular changes at the multi-omics level remain relatively limited. Using a multiomics approach, the present study identified *ACO2* as a potential candidate biomarker for AD. GEO data showed that *ACO2* expression levels in hippocampal tissue from patients with AD were significantly lower than those in healthy CTL subjects, and single-cell analysis further revealed that *ACO2* expression levels were markedly reduced in neurons from patients with AD. Subsequently, qPCR and Western blotting analyses showed significantly decreased *ACO2* mRNA and protein levels in the hippocampus of APP/PS1 mice compared to those in WT mice, and proteomic analysis showed decreased ACO2 expression levels in APP/PS1 mice. More importantly, qPCR validation results based on peripheral blood samples showed that *ACO2* mRNA expression levels were considerably lower in clinical patients than in CTL subjects. These results support the low expression level of *ACO2* in AD across multiple levels, including public databases, animal models, and clinical samples, suggesting its diagnostic value as a candidate biomarker for AD.

As a key brain region involved in memory formation and spatial orientation, the hippocampus is particularly vulnerable to damage during the progression of AD. Based on the hippocampal transcriptomic data, 20 OSRDEGs were identified using differential expression analysis, WGCNA, and cross-screening of OS-related genes. Functional analysis showed that these genes were mostly involved in processes such as aerobic respiration, the electron transport chain, ATP synthesis, oxidative phosphorylation, and redox reactions, suggesting that they may be involved in mitochondrial energy metabolism and redox homeostasis. Previous studies have shown that dysfunction of mitochondrial oxidative phosphorylation can lead to ROS accumulation, which in turn induces oxidative and neuronal damage [42,43]. Enrichment of redox enzymes and cytochrome complexes further confirmed the key role of OSRDEGs in intracellular energy conversion. Oxidoreductases and cytochrome oxidases have been shown to be important for various metabolic processes inside cells, particularly for the survival and functional maintenance of neurons [44,45]. This suggests that the OSRDEGs identified in this study may represent a group of key molecules in AD hippocampal tissue associated with mitochondrial metabolic dysfunction and OS-induced damage and that their abnormal expression may contribute to AD-related neuronal dysfunction.

Subsequently, we identified *ACO2* as a key gene using machine learning and MCODE analyses. *ACO2* belongs to the iron–sulfur [4Fe–4S] dehydrogenase family and is a key metabolic enzyme in the tricarboxylic acid (TCA) cycle [46]. Previous studies have shown that following Aβ aggregation, *ACO2* activity rapidly declines, and sustained mitochondrial oxidative stress further leads to the permanent loss of iron from its [4Fe–4S] cluster, resulting in persistent enzyme inactivation and impaired TCA cycle function [46]. Based on our bioinformatics analysis, we observed that *ACO2* expression levels in hippocampal tissue were considerably reduced in the AD group compared to the CTL group. Previous studies have shown significantly reduced *ACO2* activity in the brain tissue of patients with both familial and sporadic AD [47,48]. Another autopsy study found that *ACO2* mRNA expression levels were significantly downregulated in the hippocampal tissue of individuals with AD [49]. Previous studies have indicated a link between reduced levels of mitochondrial aconitase and the progression of AD, implying that mitochondrial aconitase dysfunction plays a role in the development of AD-related mitochondrial metabolic disorders and OS damage [50]. Notably, decreased *ACO2* levels may have dual implications in AD pathology, acting both as a consequence of oxidative injury and as a factor that may further contribute to mitochondrial dysfunction. The [4Fe–4S] cluster of *ACO2* is highly susceptible to ROS and other free radicals, and oxidative damage promotes the release of iron from this cluster, thereby impairing enzymatic activity. Longo et al. further demonstrated that exposure of neuronal cells to Aβ1–42 markedly reduced aconitase activity, suggesting that Aβ-associated oxidative stress may compromise *ACO2* function through damage to its iron–sulfur cluster [51]. Accordingly, the reduced *ACO2* activity in AD may, at least in part, reflect a downstream response to mitochondrial oxidative injury. Conversely, because *ACO2* is a key enzyme in the TCA cycle, impairment of its function may disrupt mitochondrial energy metabolism and redox homeostasis, potentially exacerbating mitochondrial dysfunction and OS [52]. These observations suggested that the relationship between *ACO2* dysregulation and oxidative stress may be bidirectional, rather than strictly unidirectional, potentially forming a self-reinforcing pathological loop. Recent animal studies have further supported the close association between oxidative stress and classical AD-like pathological changes. For example, in a D-galactose/aluminum chloride-induced AD-like rat model, Manoharan et al. observed hippocampal neuronal damage and tau hyperphosphorylation, whereas Ficus deltoidea treatment attenuated neuronal loss and reduced p-tau T181 levels, thereby providing an experimental context for the relationship between oxidative stress, tau abnormalities, and neurodegenerative injury [53]. Another study using a similar OS-driven AD-like model showed that an improvement in redox homeostasis was accompanied by the preservation of hippocampal neuronal architecture and mitochondrial ultrastructure, together with reduced lipid peroxidation and enhanced endogenous antioxidant defenses [54]. These experimental findings further support the close association among OS, mitochondrial dysfunction, tau-related alterations, and neuronal injury, providing an additional biological context for the link between reduced *ACO2* levels and mitochondrial oxidative metabolic disturbances in AD. Previous studies demonstrated that tau pathology is closely associated with impaired mitochondrial bioenergetics, increased ROS production, and mitochondrial metabolic dysfunction [55,56]. A recent systems-level review further emphasized that Aβ pathology, tau abnormalities, mitochondrial dysfunction, oxidative stress, neuroinflammation, metal dyshomeostasis, and autophagy–lysosomal dysfunction do not occur independently but instead form an interconnected and mutually reinforcing pathological network in AD [57]. Within this systems-level pathological framework and considering the established role of *ACO2* in the TCA cycle, mitochondrial function, and redox homeostasis, we speculate that *ACO2* may represent a potential mitochondrial/redox-related node within the complex pathological network of AD.

Immune cell infiltration analysis revealed a negative association between *ACO2* and M1-type macrophages. Previous studies have also reported that macrophage-derived nitric oxide can target and inhibit *ACO2*, thereby disrupting the TCA cycle and reshaping the metabolic profile of M1-type macrophages [58]. Therefore, the negative correlation observed between *ACO2* expression levels and the M1-macrophage-associated immune signature in this study may reflect an interplay between mitochondrial oxidative metabolism and immune metabolic states. From a broader perspective of neuroinflammation in AD, the recently proposed concept of the “immuno-glial connectome” emphasizes that inflammatory responses in AD are not driven by a single immune cell type, but instead, arise from a dynamic network involving microglia, astrocytes, peripheral immune cells, and blood–brain-barrier-associated processes [59]. This network may sustain and amplify neuroinflammation through cytokine signaling; OS; and pathways involving TREM2–APOE, NF-κB, JAK/STAT, and NLRP3, while also interacting with impaired Aβ clearance and tau pathology [59]. Within this framework, the M1-macrophage-associated immune signature observed in the present study may be interpreted as a transcriptional feature reflecting the inflammatory immune state of AD brain tissue. As a key mitochondrial metabolic enzyme involved in the TCA cycle and redox homeostasis, *ACO2* may be closely associated with immunometabolic alterations. Accordingly, the negative association between *ACO2* and M1 macrophage-associated immune signature further suggests a potential intersection between mitochondrial metabolism, redox imbalance, and AD-related inflammatory networks. Single-cell analysis revealed that *ACO2* expression levels in neurons from patients with AD were notably lower than those in neurons from the CTL group. Analysis of different neuronal subtypes indicated that *ACO2* expression was notably lower in both excitatory and inhibitory neurons from patients with AD than from CTL subjects.

Furthermore, differential expression of *ACO2* was validated in AD mouse models and patients. Proteomic analysis was conducted on the hippocampal tissues from APP/PS1 and WT mice, and 104 DEPs were identified. A major decrease in ACO2 expression was observed in APP/PS1 mice. Another proteomic investigation comparing hippocampal tissues from 3xTg-AD and WT mice similarly reported a marked decrease in ACO2 expression levels in the 3xTg-AD hippocampus [60]. These results indicate that ACO2 expression levels are significantly lower whether in APP/PS1 and 3xTg-AD mice than in WT mice, suggesting that the downregulation of ACO2 may be a common feature of AD. Two classic pathological hallmarks of AD, amyloid-beta plaque aggregation and abnormal hyperphosphorylation of the tau protein, can cause mitochondrial structural damage and disturb the balanced energy metabolism inside the mitochondria. As an essential functional protein participating in the TCA cycle, *ACO2* serves as a direct indicator of defective mitochondrial metabolism when its expression levels decline. Finally, *ACO2* mRNA expression levels in the peripheral blood of patients with AD and healthy CTL subjects were detected using qPCR to further evaluate its potential value as a candidate biomarker for AD. The findings showed that levels of *ACO2* mRNA in the blood were significantly in patients with AD than in healthy individuals. Previous studies have shown that *ACO2* activity is lower in the lymphocytes of patients with AD and mild cognitive impairment [50]. This further supports the potential value of *ACO2* as a biomarker of AD.

The major strength of this study lies in its “bioinformatics–model mouse–clinical sample validation” analytical framework. By integrating bulk transcriptomic, single-cell transcriptomic, and proteomic data with validation using animal models and human samples, this study achieved a high degree of consistency and reliability across different technologies, biological scales, and study populations. In addition, the application of WGCNA, machine learning algorithms, and network-based analyses substantially improved the accuracy with which key genes were identified. Nevertheless, this study has several limitations. The identification of OSRGs was mainly dependent on the GeneCards database, which may not provide a fully comprehensive gene set. Moreover, the APP/PS1 transgenic mouse model can only partially recapitulate Aβ-associated pathology and is unable to fully reproduce the multifactorial and heterogeneous pathological features observed in human AD. Second, the clinical sample size was relatively small, and independent external clinical validation was lacking. The cross-sectional design also precluded the assessment of dynamic changes in *ACO2* expression during AD progression, and all findings obtained from human samples should, therefore, be interpreted as associative rather than causal. In addition, the number of donors included in the single-cell analysis was limited, which reduced the statistical power of the donor-level comparisons. Animal experiments were also conducted using single-sex mice, which may limit the generalizability of the findings across sexes. Although decreased *ACO2* expression levels were consistently observed at both the transcript and protein levels, *ACO2* enzymatic activity was not directly measured and mitochondrial functional assessments, including mitochondrial respiration, membrane potential, and ROS levels, were not performed. Furthermore, mechanistic manipulations of *ACO2*, such as gene knockdown, overexpression, or functional rescue experiments, have not been conducted; therefore, the causal role of *ACO2* in AD-related pathological processes has not yet been established. The relatively small clinical cohort may limit the generalizability and statistical robustness of our findings. Peripheral blood *ACO2* levels may also be influenced by systemic or metabolic conditions, which are potential confounding factors. Therefore, future studies using larger multicenter clinical cohorts, including both sexes in animal experiments, and direct mechanistic and mitochondrial functional assays are needed to further validate these findings.

## 5. Conclusions

This study integrated transcriptomic and scRNA-seq data from patients with AD and revealed that *ACO2* was significantly downregulated in the hippocampal tissues of patients with AD. In addition, animal experiments showed that both *ACO2* mRNA and protein levels were significantly reduced in the hippocampal tissues of APP/PS1 mice compared to those in WT mice. Consistently, clinical qPCR validation further demonstrated that *ACO2* mRNA expression levels in the peripheral blood of patients with AD was significantly lower than that in the peripheral blood of healthy CTL subjects. Taken together, these findings from multiple perspectives, including public data analysis, single-cell analysis, animal models, and clinical sample validation, indicate that *ACO2* may exhibit a stable low expression level pattern in AD. These results provide new insights into the potential role of *ACO2* in the pathological progression of AD and suggest that *ACO2* may have research value as a candidate biomarker for AD.

## Data Availability

The original contributions presented in this study are included in the article and Supplementary Materials. The proteomic data have been included in Supplementary File S1 (Table S11). Further inquiries can be directed to the corresponding authors.

## Supplementary Materials

The following supporting information can be downloaded at: https://www.mdpi.com/article/10.3390/biom16101435/s1, Supplementary File S1: Tables S1–S11. Supplementary File S2: Figures S1–S4, Tables S12–S14.
