# Supplementary material for: Integrated Multi-Omics Analysis Reveals ACO2 as an Oxidative-Stress-Related Biomarker in Alzheimer’s Disease
Source: Biomolecules. 2026 Oct 2;16(10):1435. doi: 10.3390/biom16101435 (PMC13702750; doi:10.3390/biom16101435)
Supplement: Supplementary file 1 [file biomolecules-16-01435-s001.zip › Supplementary file S2.pdf]

Sample preparation

The mouse hippocampus was ground it into powder under liquid nitrogen. 1.5 mL powder was taken and added with lysis buffer (containing 8 M urea, 1 mM PMSF, and 2 mM EDTA). The mixture was then put on ice for 5 minutes, before being spun at 4°C and 15,000 g for 10 minutes. The supernatant was collected to determine protein concentration using a special kit called the BCA kit. According to the measured protein levels, 100 μg of the sample was taken and adjusted to a final volume of 200 μL using 8 M urea. Subsequently, DTT (final concentration 5 mM) was added and maintained at 37°C for 45 min. Then iodoacetamide (final concentration 11 mM) was added to alkylate at room temperature in the dark for 15 min. Following the alkylation process, a volume of 800 μL of a 25 mM ammonium bicarbonate solution and 2 μL of trypsin are required (Promega, Cat. No. V5280) were added, and the mixture was incubated overnight at 37 °C for digestion. The pH of the digested peptides was adjusted to 2-3 using 20% TFA, then the sample underwent desalting using C18 resin (Millipore, Billerica, MA). The determination of peptide concentration was finally achieved through the utilization of the Pierce™ Quantitative Peptide Assay Kit (Thermo Fisher) with established standards.

LC-MS/MS analysis

Proteomics was carried out with a Vanquish Neo UHPLC nanoliter-scale liquid chromatography system. Mobile phase A consisted of 0.1% formic acid in water, while mobile phase B contained 0.1% formic acid in acetonitrile. A capture-analysis dual-column method was adopted, with the trap column being a PepMap Neo Trap Cartridge (300 μm × 5 mm, 5 μm) and the analytical column being an Easy-Spray™ PepMap™ Neo UHPLC column (150 μm × 15 cm, 2 μm). The analytical column was set at 55°C, with a flow rate of 2.5 μL/min, an injection volume of 200 ng, a gradient time of 6.9 minutes, and a total run time of 8 minutes. The Orbitrap Astral high-resolution mass spectrometer (Thermo Scientific) was used for DIA analysis in positive ion mode. The precursor ion scan range was 380-980 m/z, with the primary mass spectrometer resolution is 240,000 at 200 m/z, a Normalized AGC Target of 500%, and a Maximum IT of 5 ms. For MS2, we used a DIA data acquisition mode, with 299 scan windows, an Isolation Window of 2 Th, an HCD

Collision Energy of 25%, a Normalized AGC Target of 500%, and a Maximum IT of 3 ms.

Proteomic Data Processing and Statistical Analysis

A total of eight independent biological samples were included in the proteomic analysis, comprising hippocampal samples from four APP/PS1 mice and four WT mice. DIA mass spectrometry raw data were analyzed for protein identification and quantification using DIA-NN software in library-free mode against the UniProt mouse proteome database (uniprotkb_proteome_UP000000589_mouse_54910_20240528.fasta; 54,910 protein sequences). Deep-learning-based spectral prediction was enabled, together with the Match Between Runs (MBR) function, to generate a spectral library from the DIA data and subsequently reanalyze the dataset. Identifications at both the precursor ion and protein levels were filtered at a false discovery rate (FDR) of 1%. Protein quantification was performed using the MaxLFQ algorithm implemented in DIA-NN, and protein intensity values for each sample were extracted from the search results. Sample-wise median normalization was then applied by dividing the intensity of each protein by the median intensity of all quantified proteins within the corresponding sample, thereby reducing the influence of overall signal-intensity differences among samples. For comparisons between the WT and APP/PS1 groups, fold change (FC) was calculated as the ratio of the mean normalized protein abundance across biological replicates in the two groups, and an independent-samples t-test was used to obtain nominal P values.

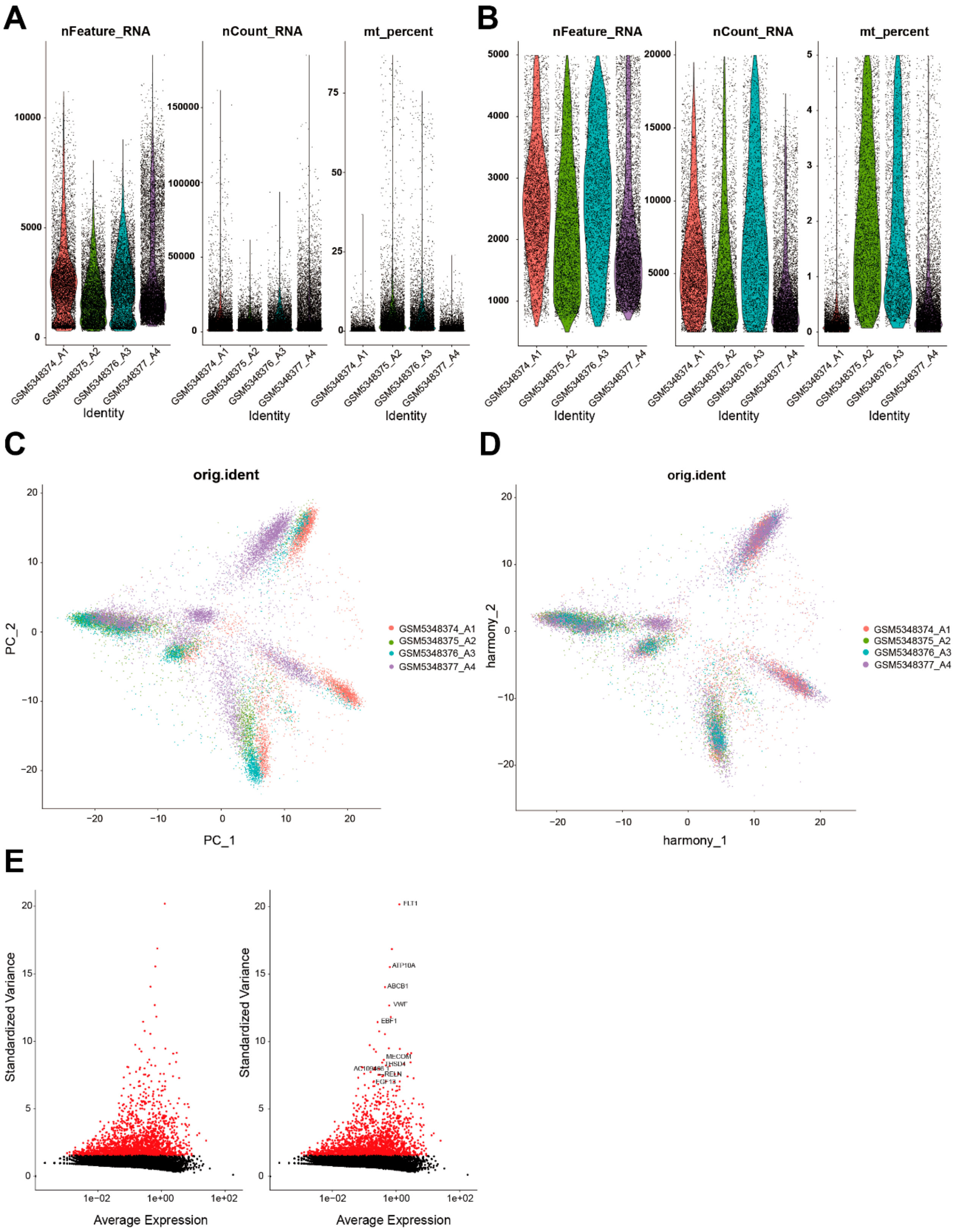

Figure S1. Single-cell analysis. (A) Violin plot before quality control. (B) Violin plot after quality control. (C) PCA plot before batch effect removal. (D) PCA plot after batch effect removal. (E) ACO2 expression levels in excitatory neurons of AD and CTL.

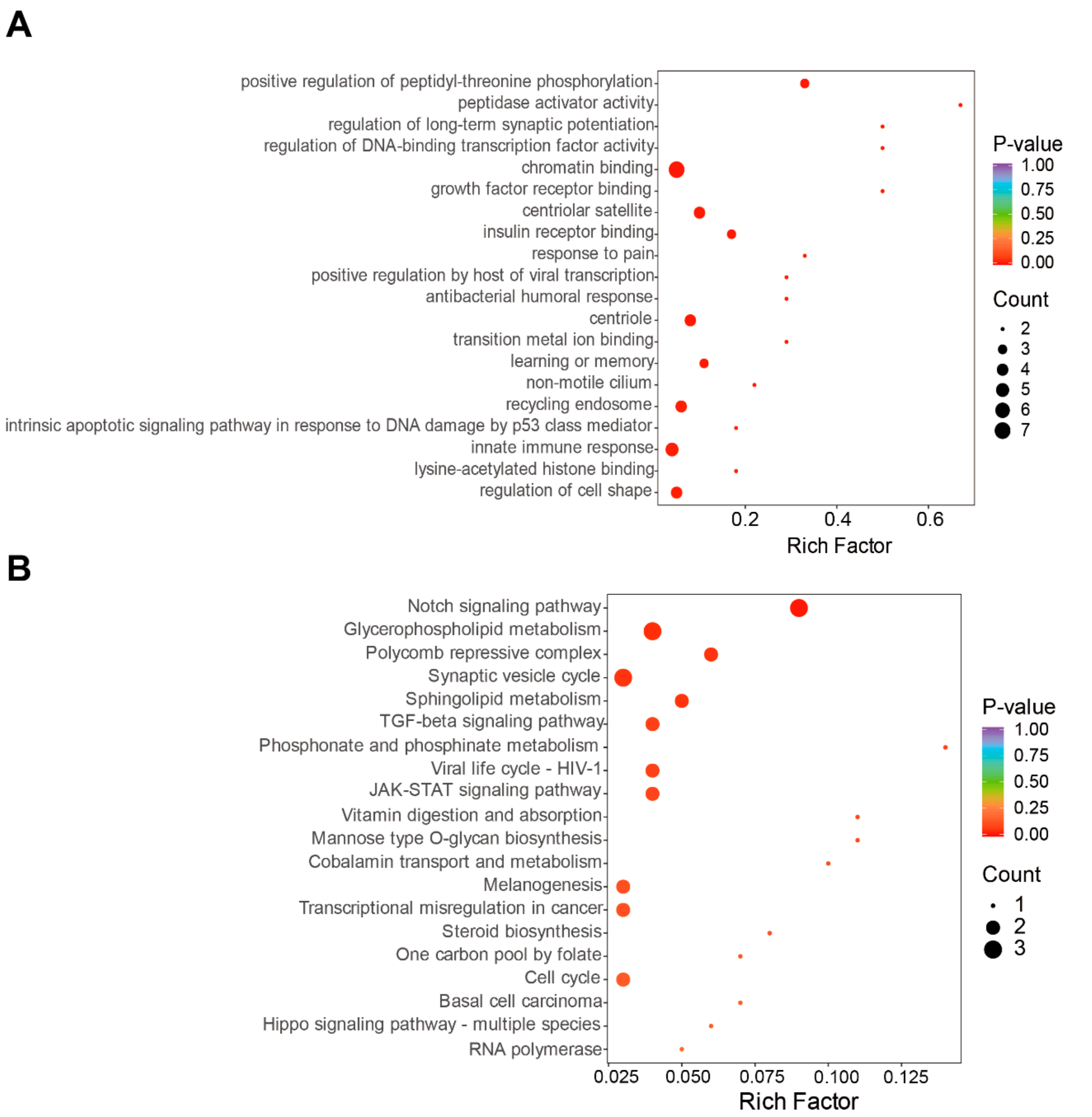

Figure S2. Enrichment Analysis of Differentially Expressed Proteins. (A) Functional categories of the differentially expressed proteins identified through GO analysis. (B) Pathway characterization of the differentially expressed proteins using KEGG annotations.

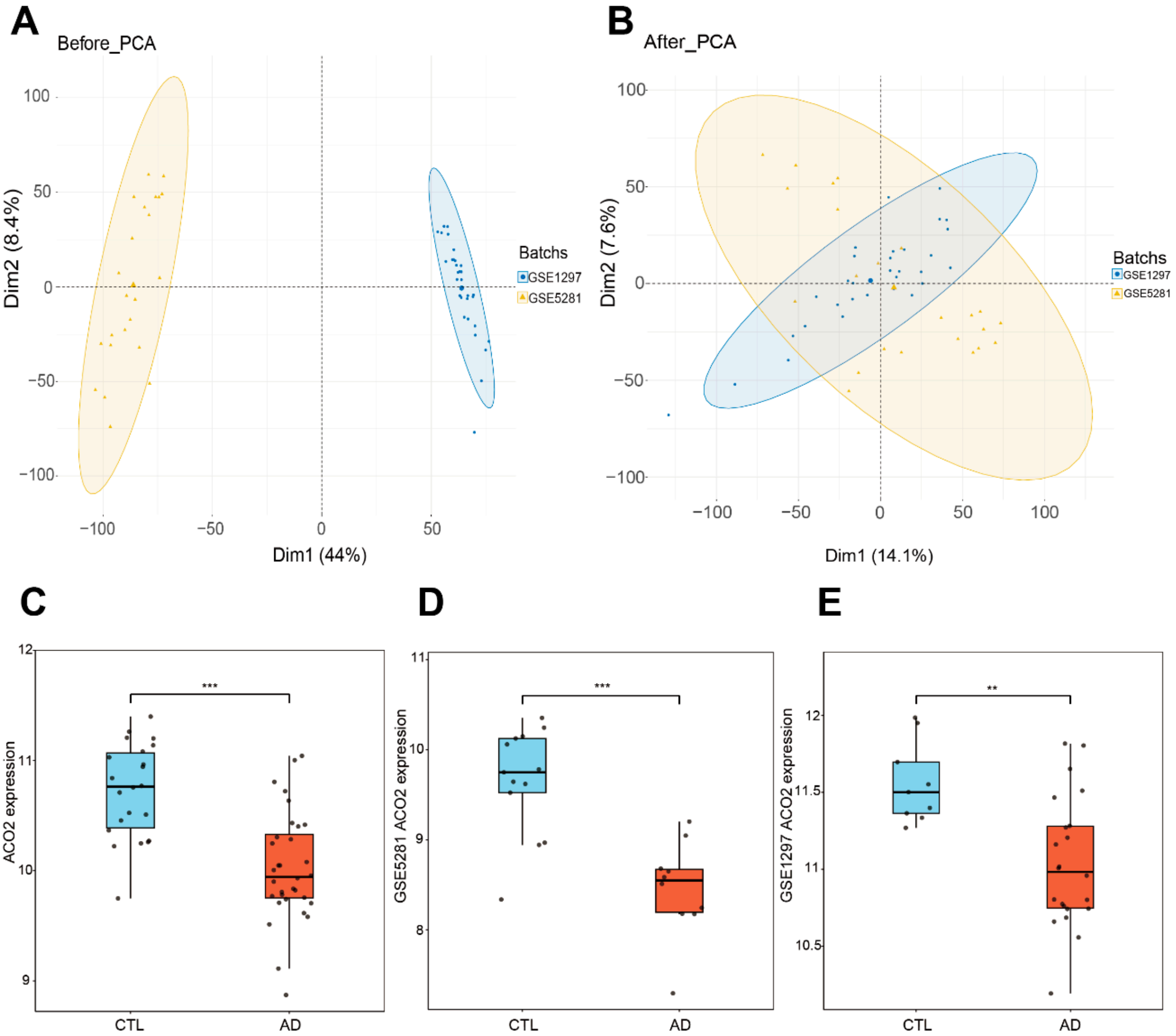

Figure S3. Batch-effect correction of GSE5281 and GSE1297 and sensitivity analysis of ACO2 expression. (A) PCA of the GSE5281 and GSE1297 datasets before batch-effect correction, showing clear separation between samples from the two datasets. (B) PCA after batch-effect correction using the ComBat method, showing increased overlap between samples from the two datasets. (C) ACO2 expression in the integrated dataset after ComBat correction (CTL, $n = 22$; AD, $n = 32$). (D) ACO2 expression in GSE5281 analyzed independently (CTL, $n = 13$; AD, $n = 10$). (E) ACO2 expression in GSE1297 analyzed independently (CTL, $n = 9$; AD, $n = 22$). ACO2 showed a consistent decrease in AD samples in both independent datasets, in agreement with the expression pattern observed in the ComBat-corrected integrated dataset, suggesting that the observed ACO2 expression change was not driven solely by a single dataset, microarray platform, or batch-correction approach. Box plots show the median and interquartile range, and each point represents an individual sample. $^{**}p < 0.01$ and $^{***}p < 0.001$. CTL, control; AD, Alzheimer's disease; PCA, principal component analysis.

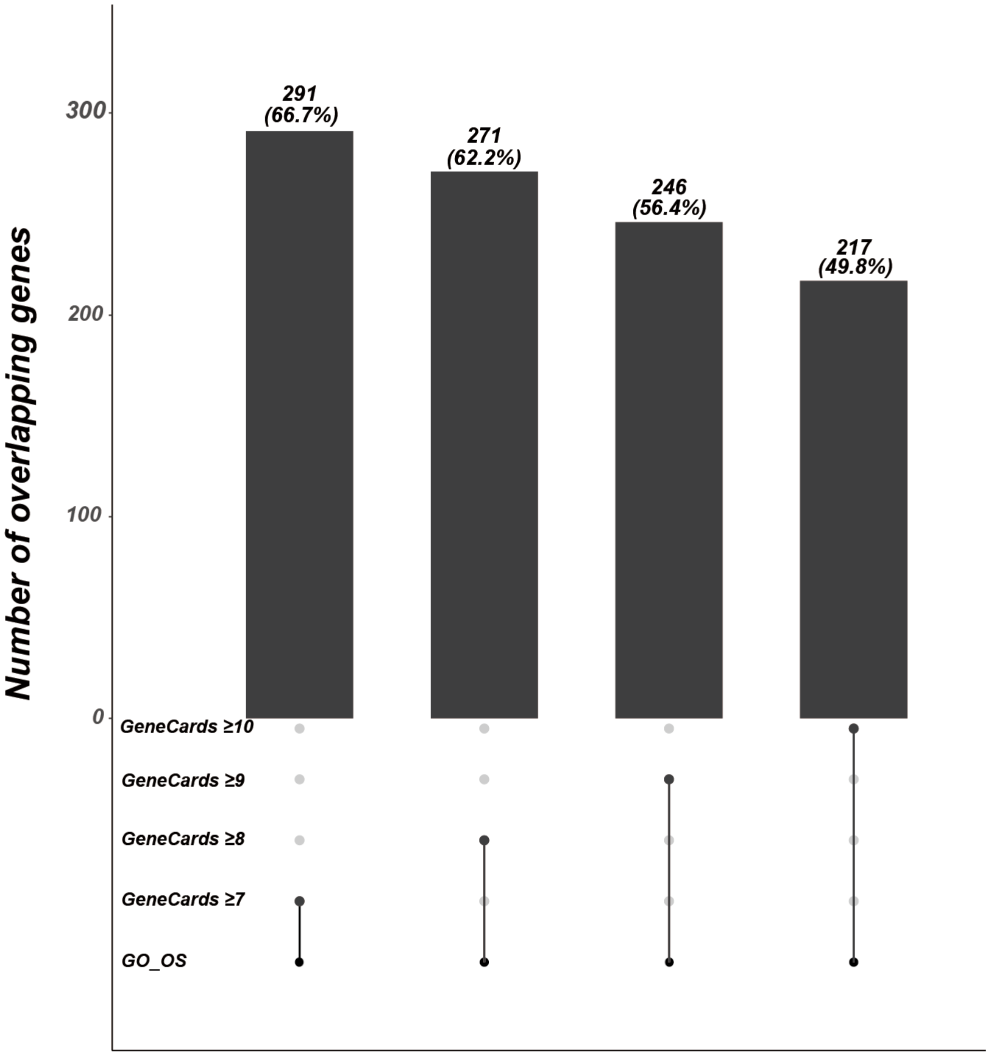

Figure S4. Overlap between GeneCards-derived gene sets at different relevance score thresholds and the GO oxidative stress gene set. GO:0006979 was used as a standardized oxidative stress reference gene set. The overlaps between this reference set and GeneCards-derived gene sets at relevance score thresholds of ≥7, ≥8, ≥9, and ≥10 were evaluated. The corresponding numbers of overlapping genes were 291, 271, 246, and 217, representing 66.7%, 62.2%, 56.4%, and 49.8% of the GO:0006979 gene set, respectively. The coverage of standardized oxidative stress-related genes progressively decreased as the GeneCards screening threshold became more stringent.

Table S12. Sequencing of primer transcripts.

Primer names	Gene sequence（5′ to 3′）
Mus ACO2 F	CACGTCACTTTCATGCGACC
Mus ACO2 R	TCGGATGTACTCACTGGGCT
Mus GAPDH F	AACTTTGGCATTGTGGAAGGG
Mus GAPDH R	GACACATTGGGGGTAGGAACA
Hum ACO2 F	GTGATTGGCGTGAAGCTGAC
Hum ACO2 R	AAGGGAACACGGAAGTGGTG
Hum GAPDH F	GGAGCGAGATCCCTCCAAAAT
Hum GAPDH R	GGCTGTTGTCATACTTCTCATGG

Table S13. Reagent Information.

Reagent Name (Abbreviation)	Reagent Name (Full)	Supplier
BSA	Albumin from bovine serum	Wuhan Chucheng Zhengmao Technology Co., Ltd.
DTT	DL-Dithiothreitol	Solarbio
EDTA	Ethylene Diamine Tetraacetic Acid	Sinopharm
G250	Xylene brilliant cyaninG	Sinopharm
IAM	Iodoacetamide	Aladdin
PMSF	Phenylmethanesulfonyl fluoride	Xiya Reagent
SDS	Sodium dodecyl sulfate	Sinopharm
TEAB	Tetraethylammonium bromide	Sigma
Thiourea	Thiourea	Sinopharm
Tris	Tris	Solarbio
Trypsin	Trypsin	Promega
Urea	Urea	Sigma
Protein marker	/	Fermentas
Acetone	/	Sinopharm
BCA assay kit	/	Beyotime Biotechnology

Table S14. Instrument Information.

Instrument Name	Supplier	Model
Ultrasonic Cell Disruptor	Ningbo Scientz Biotechnology Co., Ltd.	JY92-11N
Benchtop High-Speed Refrigerated Centrifuge	Hunan Xiangyi Laboratory Instrument Development Co., Ltd.	TGL-20M
Freeze Dryer	Ji Aimu	CV 600
Electrophoresis System	Beijing Liuyi Instrument Factory	DYY-6C
Electrophoresis Tank	Beijing Liuyi Instrument Factory	DYCZ-24DN
Microplate Reader	Molecular Devices LLC	Cmax Plus
Mass Spectrometer	Thermo Fisher Scientific	Orbitrap™ Astral™
Pipette	Eppendorf	/
